# Characterizing the normal proteome of human ciliary body

**DOI:** 10.1186/1559-0275-10-9

**Published:** 2013-08-01

**Authors:** Renu Goel, Krishna R Murthy, Srinivas M Srikanth, Sneha M Pinto, Mitali Bhattacharjee, Dhanashree S Kelkar, Anil K Madugundu, Gourav Dey, Sujatha S Mohan, Venkatarangaiah Krishna, TS Keshava Prasad, Shukti Chakravarti, HC Harsha, Akhilesh Pandey

**Affiliations:** 1 Institute of Bioinformatics, International Technology Park, Bangalore 560 066, India; 2 Department of Biotechnology, Kuvempu University, Shankaraghatta, Shimoga 577 451, Karnataka, India; 3 Amrita School of Biotechnology, Amrita Vishwa Vidyapeetham, Kollam 690 525, Kerala, India; 4 Vittala International Institute Of Ophthalmology, Bangalore 560 085, Karnataka, India; 5 Manipal University, Madhav Nagar, Manipal 576104, Karnataka, India; 6 Research Unit for Immunoinformatics, RIKEN Research Center for Allergy and Immunology, RIKEN Yokohama Institute, Kanagawa 230 0045, Japan; 7 Centre of Excellence in Bioinformatics, Bioinformatics Centre, School of Life Sciences, Pondicherry University, Puducherry 605 014, India; 8 Johns Hopkins University School of Medicine, Baltimore 21205, MD, USA; 9 Department of Cell Biology, Johns Hopkins School of Medicine, Baltimore, MD, USA; 10 Department of Ophthalmology, Johns Hopkins School of Medicine, Baltimore, MD, USA; 11 McKusick-Nathans Institute of Genetic Medicine, Departments of Biological Chemistry, Oncology and Pathology, Johns Hopkins University School of Medicine, Baltimore 21205, MD, USA

**Keywords:** Aqueous humor, Proteome discoverer, Protein biomarkers, Ciliary processes, Non-pigmented epithelial layer, Pigmented epithelial layer, Cyclitis and glaucoma

## Abstract

**Background:**

The ciliary body is the circumferential muscular tissue located just behind the iris in the anterior chamber of the eye. It plays a pivotal role in the production of aqueous humor, maintenance of the lens zonules and accommodation by changing the shape of the crystalline lens. The ciliary body is the major target of drugs against glaucoma as its inhibition leads to a drop in intraocular pressure. A molecular study of the ciliary body could provide a better understanding about the pathophysiological processes that occur in glaucoma. Thus far, no large-scale proteomic investigation has been reported for the human ciliary body.

**Results:**

In this study, we have carried out an in-depth LC-MS/MS-based proteomic analysis of normal human ciliary body and have identified 2,815 proteins. We identified a number of proteins that were previously not described in the ciliary body including importin 5 (*IPO5*), atlastin-2 (*ATL2*), B-cell receptor associated protein 29 (*BCAP29*), basigin (*BSG*), calpain-1 (*CAPN1*), copine 6 (*CPNE6*), fibulin 1 (*FBLN1*) and galectin 1 (*LGALS1*). We compared the plasma proteome with the ciliary body proteome and found that the large majority of proteins in the ciliary body were also detectable in the plasma while 896 proteins were unique to the ciliary body. We also classified proteins using pathway enrichment analysis and found most of proteins associated with ubiquitin pathway, EIF2 signaling, glycolysis and gluconeogenesis.

**Conclusions:**

More than 95% of the identified proteins have not been previously described in the ciliary body proteome. This is the largest catalogue of proteins reported thus far in the ciliary body that should provide new insights into our understanding of the factors involved in maintaining the secretion of aqueous humor. The identification of these proteins will aid in understanding various eye diseases of the anterior segment such as glaucoma and presbyopia.

## Background

**Figure 1:**
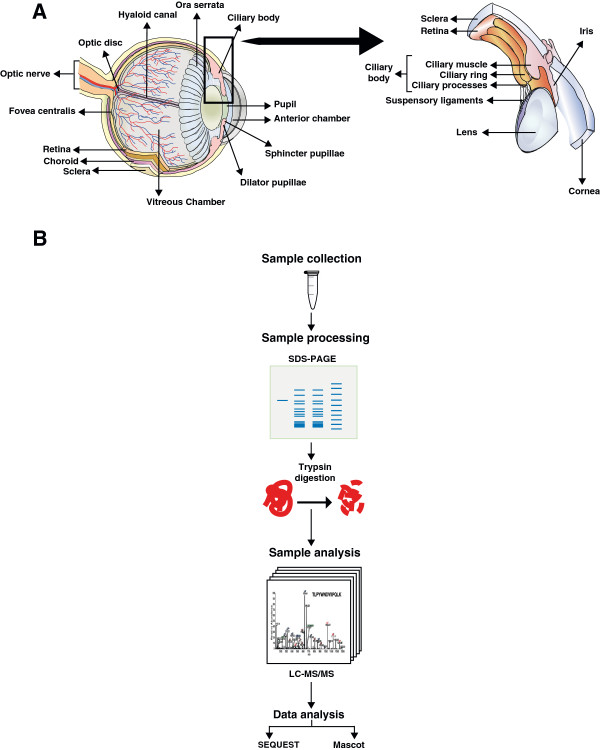
**Schematic structure of the eye and experimental strategy for proteomic analysis of human ciliary body. Panel A.** shows anatomy of the eye with a zoomed in view of the ciliary body. **Panel B.** depicts the proteomic workflow employed for the study.

The ciliary body, iris and choroid comprise the vascular uveal coat of the eye. The ciliary body forms a ring along the inner wall of the globe and extends from the iris anteriorly to the ora serrata posteriorly as shown in Figure 1A. It is predominantly made up of smooth muscle that is arranged in longitudinal radial and circular fashion. The ciliary body is composed of the ciliary muscle and ciliary processes. Ciliary processes are approximately 70 in number in humans and project inwards as radial ridges [1]. The ciliary body is highly vascular and supplied by the anterior ciliary and long posterior ciliary vessels [2,3]. The ciliary processes consist of a central core of connective tissue stroma which is covered by a double layered epithelium. The inner non-pigmented epithelial layer is in direct contact with the aqueous [4]. It is formed by a layer of columnar cells which contain numerous mitochondria, rough and smooth endoplasmic reticulum which is characteristic of metabolically active cells. The outer-pigmented epithelial cell layer is a layer of cuboidal cells which are abundant in melanosomes that are relatively poor in intracellular organelles. It lies between the non-pigmented epithelial layer and the connective tissue stroma [1].

The non-pigmented epithelial layer of the ciliary body secretes aqueous humor by a process of active transport, through diffusion and ultrafiltration [5]. The aqueous humor bathes the avascular structures of the eye such as the crystalline lens, posterior surface of the cornea, the anterior vitreous and the trabecular meshwork before exiting the eye through the canal of Schlemm into the episcleral veins. A small fraction of the aqueous also exits the eye between the muscle bundles of the ciliary body to the supraciliary and suprachoroidal spaces, commonly called the uveoscleral pathway [5]. This constant flow of aqueous replenishes the nutrients required for these avascular tissues and carries away their metabolic wastes. The aqueous humor dynamics also helps to maintain the intraocular pressure of the eye that is essential for maintaining the optical and refractive properties of the eye [6,7]. The ciliary muscles contract, the zonules relax and the lens becomes thicker for near vision while distant vision involves relaxation of the ciliary muscles, contraction of the zonules and thinning of the lens. The ciliary body functions are implicated in ophthalmic pathology such as open and closed angle glaucoma [8], due to a complex imbalance in aqueous humor production and drainage, cyclitis or inflammation of the ciliary body and presbyopia, which is characterized by a diminution of the ability of the eye to accommodate [9-11].

Identification of the protein constituents of tissues can lead to a better understanding of their normal physiology. Previous molecular analysis of the ciliary body has provided some insights into the expression profiles of the two ciliary epithelia. The majority of proteomic studies of the human ciliary body reported to date are based on immunohistochemistry, Western blot or immunofluorescence-based studies. Wu *et al*. identified nitric oxide synthase 1 neuronal (NOS1), NOS2 and NOS3 by Western blot and immunohistochemistry [12]. Flugel-Koch *et al*., identified tyrosine hydroxylase, neuropeptide Y, tachykinin, NOS1, solute carrier family 18 member 3, calbindin 2, calcitonin-related polypeptide alpha, 2,4-dienoyl CoA reductase 1 mitochondrial by immunohistochemical assays [13]. Pattwell *et al*., identified enolase 2, opticin (*OPTC*), S100 calcium binding protein B, vimentin and collagen type II alpha 1 (*COL2A1*) by immunofluorescence assays [14]. Although, proteomic approaches have been used to identify proteins in eye tissues such as vitreous, aqueous humor and retina, to the best of our knowledge, a comprehensive analysis of proteome of the ciliary body has not yet been carried out. In this study, we report a comprehensive catalogue of proteins expressed in the normal ciliary body and provide the subcellular localization, molecular function and biological processes associated with these proteins. This characterization of the ciliary body proteome from healthy individuals may serve as a valuable template to compare the ciliary body proteomic changes occurring in other sight-threatening pathological conditions such as glaucoma and macular degeneration.

## Results and discussion

Proteomic analysis of the ciliary body samples was carried out by digestion of bands excised from an SDS-PAGE gel as illustrated in the Figure 1B. MS/MS analysis was carried out for 30 in-gel digested fractions on an LTQ-Orbitrap Velos ETD mass spectrometer. The corresponding MS data were searched using two different search algorithms – Mascot and SEQUEST - against the NCBI RefSeq human protein database 50 (N=33,832 proteins) with known contaminants. MS/MS spectra resulted in identification of 157,782 peptide-spectral matches (PSM) and these PSM were filtered for first rank assignment that passed 1% FDR threshold. In total 19,547 unique peptides sequences were identified and these peptides resulted in identification of 2,815 proteins. A complete list of proteins identified in the ciliary body is provided in Additional file 1: Table S1 along with unique number of peptides, spectrum count, sequence coverage, intensity based absolute quantification (iBAQ) score, subcellular localization, molecular function, biological process and domains/motifs. A non-redundant list of peptides identified from this study is provided in Additional file 2: Table S2.

### Proteins previously described in the ciliary body

**Figure 2:**
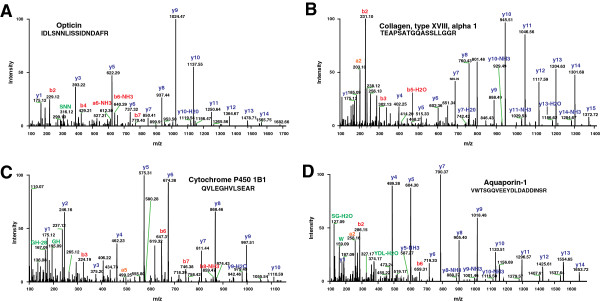
**MS**/**MS spectra of previously described proteins. A**. The peptide IDLSNNLISSIDNDAFR from opticin **B**. shows the MS/MS spectra of peptide TEAPSATGQASSLLGGR from collagen, type XVIII, alpha 1 **C**. The Peptide QVLEGHVLSEAR belongs to cytochrome p450 1B1 **D**. VWTSGQVEEYDLDADDINSR peptide from aquaporin 1.

Among the identified proteins, we found a number of proteins that had been previously described in the ciliary body, confirming the validity of our proteomic approach. A search of the published literature resulted in <50 proteins that have been reported in the human ciliary body to date. Many groups using different techniques as summarized in Table 1 identified these as individual proteins based on targeted molecules of interest. Among the proteins previously shown to be in the ciliary body are collagen type XVIII alpha 1 (*COL18A1*), cytochrome P450 family 1 subfamily B polypeptide 1 (*CYP1B1*), Opticin (*OPTC*) and aquaporin 1 (*AQP1*). Representative MS/MS spectra of these identified proteins in this study are shown in Figure 2.

**Table 1:** A summary of published studies for the ciliary body protein identification

	**Study**	**Method**	**Source**	**Proteins identified**
1	Wu *et al*. [12]	Immunohistochemistry, Western blot	PCE and NPCE cells	NOS1
2	Wu *et al*. [12]	Immunohistochemistry, Western blot	PCE and NPCE cells	NOS2
3	Wu *et al*. [12]	Immunohistochemistry, Western blot	PCE and NPCE cells	NOS3
4	Krueger *et al*. [59]	Immunohistochemistry	PCE and NPCE cells	SCNN1B
5	Krueger *et al*. [59]	Immunohistochemistry	PCE and NPCE cells	SCNN1G
6	Gabriel *et al*. [60]	Immunohistochemistry, Western blot	Ciliary body	ADAMTSL4
7	Yamanouchi *et al*. [61]	Immunofluorescence, immunohistochemical assay	NPCE	FBN1
8	Yamanouchi *et al*. [61]	Immunofluorescence, immunohistochemical assay	NPCE	FBN2
9	Chowdhury *et al*. [62]	Immunohistochemistry, Western blot	Ciliary body	SPP1
10	Russell-Randall *et al*. [63]	Immunofluorescence	NPCE	OPRK1
11	Zhang *et al*. [64]	immunofluorescence	NPCE	BEST2
12	Kraft *et al*. [65]	Immunofluorescence	PCE and NPCE cells	SLCO2A1
13	Kraft *et al*. [65]	Immunofluorescence	PCE and NPCE cells	SLCO2B1
14	Wang *et al*. [66]	Immunohistochemistry	NPCE	COX2
15	Flugel-Koch *et al*. [13]	Immunohistochemical	Ciliary body	TH
16	Flugel-Koch *et al*. [13]	Immunohistochemical	Ciliary body	NPY
17	Flugel-Koch *et al*. [13]	Immunohistochemical	Ciliary body	TAC1
18	Flugel-Koch *et al*. [13]	Immunohistochemical	Ciliary body	SLC18A3
19	Flugel-Koch *et al*. [13]	Immunohistochemical	Ciliary body	NOS1
20	Flugel-Koch *et al*. [13]	Immunohistochemical	Ciliary body	CALB2
21	Flugel-Koch *et al*. [13]	Immunohistochemical	Ciliary body	CALCA
22	Flugel-Koch *et al*. [13]	Immunohistochemical	Ciliary body	DECR1
23	Yucel *et al*. [67]	Immunofluorescence	Ciliary body	PDPN
24	Yucel *et al*. [67]	Immunofluorescence	Ciliary body	LYVE1
28	Mao *et al*. [68]	Immunohistochemical	NPCE cells	SH3PXD2B
29	Ooi *et al*. [69]	Western blot, Immunofluorescence	Ciliary body	ADRA2A
30	Webb *et al*. [70]	Immunofluorescence and immunoblot	NPCE	KLK1
31	Webb *et al*. [70]	Western blot	Ciliary body	BDKRB1
32	Webb *et al*. [70]	Western blot	Ciliary body	BDKRB2
33	Pattwell *et al*. [14]	Immunofluorescence	NPCE	Opticin
34	Pattwell *et al*. [14]	Immunofluorescence	NPCE	COL2A1
35	Pecorella *et al*. [71]	Immunohistochemistry	Ciliary body	ENO2
36	Pecorella *et al*. [71]	Immunohistochemistry	Ciliary body	S100B
37	Pecorella *et al*. [71]	Immunohistochemistry	Ciliary body	VIM
38	Marmorstein *et al*. [72]	Immunofluorescence	NPCE	BEST2
39	Ostojic *et al*. [73]	Immunofluorescence	NPCE	CYGB
40	Ostojic *et al*. [73]	Immunofluorescence	NPCE	NGB
41	Assheton *et al*. [74]	Immunofluorescence	NPCE	Opticin
42	Toyran *et al*. [75]	Immunohistochemistry	NPCE	BMP7
43	Toyran *et al*. [75]	Immunohistochemistry	NPCE	GDF5
44	Maatta *et al*. [20]	Immunohistochemistry	PCE and NPCE cells	COL18A1
45	Ohlmann *et al*. [21]	Immunohistochemistry	NPCE	COL18A1
46	Ramesh *et al*. [17]	Immunolocalisation	Ciliary body	Opticin
47	Ramesh *et al*. [17]	Immunolocalisation	Ciliary body	COL18A1
48	Bishop *et al*. [76]	in situ hybridisation	NPCE	Opticin
49	Wollensak *et al*. [77]	Immunohistochemistry	Ciliary body	ECE1
50	Doshi *et al*. [23]	Immunoreactivity	NPCE	CYP1B1
51	Sonsino *et al*. [78]	Immunofluorescence	NPCE	TJP1
52	Sonsino *et al*. [78]	Immunofluorescence	NPCE	OCLN
53	Hamann *et al*. [79]	Immunohistochemistry	Ciliary body	AQP1
54	Wang *et al*. [80]	Immunoreactivity	Ciliary body	LAMB1
55	Wang *et al*. [80]	Immunoreactivity	Ciliary body	LAMB2
56	Kumagai *et al*. [81]	Immunohistochemistry	NPCE	SLC2A1
57	Peress *et al*. [82]	Immunohistochemical	PCE and NPCE cells	TGFB2
58	Peress *et al*. [82]	Immunohistochemical	PCE and NPCE cells	TGFB3
59	Wistrand *et al*. [83]	Immunofluorescence	PCE and NPCE cells	CA2

Opticin is associated with the extracellular matrix and belongs to leucine-rich repeat protein family [15]. It is also abundantly expressed in other parts of the eye including the vitreous humor, cornea, iris and retina [15-18]. *OPTC* has been reported as a candidate gene for primary open angle glaucoma [19]. Collagen alpha-1(XVIII) (*COL18A1*) is expressed in both pigmented and non-pigmented epithelial layer cells and confirmed by immunohistochemistry [17,20,21]. It is an extracellular matrix protein with collagen and thrombospondin like domains and releases endostatin multiple biological activities. Endostatin is a proteolytic fragment of collagen XVIII, released from its C-terminal end, and inhibits endothelial cell proliferation, tumorigenesis and angiogenesis [22].

CYP1B1 is a member of the cytochrome P450 superfamily of enzymes. Doshi *et al*. have shown expression of CYP1B1 in non-pigmented epithelial layer by immunoreactivity screening [23]. CYP1B1 is expressed in fetal eyes and plays a vital role in morphogenesis of iris and ciliary body [24]. Aquaporins are integral membrane proteins that function as molecular water channel proteins. These proteins have pores through which water crosses the plasma membranes of various human tissues. In the eye, water homeostasis is essential for protecting the epithelium, and maintaining ocular transparency for optimal vision [25]. The sodium/potassium transporting ATPase subunit activates Na+ and K+ located in the ciliary body to recruit energy required for transport by hydrolysis of adenosine triphosphate to adenosine diphosphate [26]. We identified AQP1 that play a role in the production of aqueous humor in the ciliary body epithelia and movement of aqueous humor into the anterior chamber of the eye [27].

### Novel proteins identified in the ciliary body

**Figure 3:**
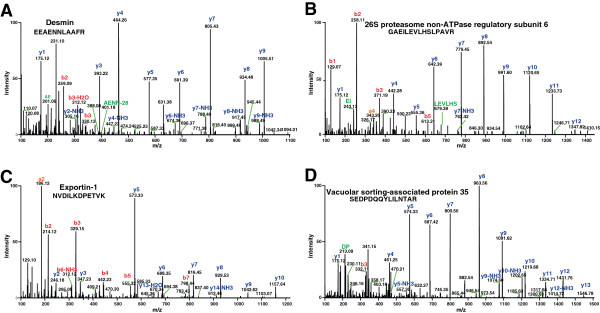
**MS**/**MS spectra of novel proteins identified. A**. shows the MS/MS spectra of peptide, EEAENNLAAFR, from Desmin **B**. The peptide, GAEILEVLHSLPAVR, derived from 26S proteasome non-ATPase regulatory subunit 6 **C**. NVDILKDPETVK Peptide from exportin-1 **D**. SEDPDQQYLILNTAR Peptide from vacuolar sorting-associated protein 35.

The majority of identified proteins were not previously reported in the ciliary body proteome. A partial list of these proteins is provided in Table 2. Representative MS/MS spectra of four proteins identified in this study - desmin, 26S proteasome non-ATPase regulatory subunit 6, exportin 1 and vacuolar protein sorting-associated protein 35 are shown in Figure 3 and described in the subsequent sections.

**Table 2:** A partial list of novel proteins identified in this study

**Gene symbol**	**Protein**	**Subcellular component**	**Biological process**	**Molecular function**
P4HB	Calpain small subunit 1	Endoplasmic reticulum	Protein metabolism	Isomerase activity
*GLG1*	Golgi apparatus protein 1	Golgi apparatus	Biological_process unknown	Receptor binding
*CD47*	Leukocyte surface antigen CD47	Cell surface, Plasma membrane	Immune response	Molecular function unknown
*KDELR2*	ER lumen protein retaining receptor 2	Endoplasmic reticulum	Transport	Transporter activity
*NCF2*	Neutrophil cytosol factor 2	Cytoplasm	Metabolism, Energy pathways	Catalytic activity
*PFN1*	Profilin-1	Cytoplasm	Cell growth	Cytoskeletal protein binding
*MDH2*	Myosin-11	Mitochondrion	Metabolism, Energy pathways	Catalytic activity
*CBR1*	Pyruvate kinase isozymes M1/M2	Cytoplasm	Metabolism, Energy pathways	Oxidoreductase activity
*APOA1*	Tubulin beta-2A chain	Extracellular	Transport	Transporter activity
*HSPA5*	78 kDa glucose-regulated protein	Endoplasmic reticulum	Protein metabolism	Chaperone activity
*EEF1A1*	Elongation factor 1-alpha 1	Cytoplasm	Regulation of cell cycle	Transcription regulator activity
*DDAH1*	Alpha-1-antitrypsin	Cytoplasm	Metabolism, Energy pathways	Hydrolase activity
*C3*	Complement C3	Extracellular	Immune response	Complement activity
*RAB1A*	NADH-ubiquinone oxidoreductase 75 kDa subunit, mitochondrial	Golgi apparatus	Cell communication, Signal transduction	GTPase activity

Vesicle mediated protein sorting (VPS) family plays a significant role in separation of intracellular molecules into different organelles. VPS1 to VPS40 proteins are involved in the recycling of membrane-associated proteins and retrograde transport of molecules from endosomes to the trans-golgi network. The heteropentameric retromer system consists of dimers of *SNX1*, *SNX2*, *SNX5*, *SNX6*, and a heterotrimer of vacuolar protein sorting-associated protein 26 (*VPS26*), *VPS29*, *VPS35 *[28]. Sorting nexin dimer is essential for the employment of retromer to the endosomal membrane, and VPS proteins assist in the cargo recognition. Interestingly, we found most of the molecules listed in intracellular trafficking and protein sorting mechanisms in our study given in Additional file 1: Table S1.

Desmin is a muscle specific class III intermediate filament which connects myofibrils to the plasma membrane. Mutations in the Desmin gene are associated with desmin related myopathy which affects cardiac, skeletal, and smooth muscle [29]. It should be further studied to see the role in the ciliary body. Karyopherin family proteins involved in transporting molecules between the cytoplasm and the nucleus and transport occurs through the nuclear pore. It mediates nuclear import and export of ribosomal proteins required for ribosome biogenesis. Molecules transport occurs across the nuclear envelope through importins and exportins proteins. Both proteins are regulated by the small GTPase Ran and localized to nucleus, cytoplasm, nucleolus, kinetochore and cytosol [30-32]. Importins identify their substrates in the cytoplasm and transport them to the nucleus. Here, the cargo is released by binding of RanGTP to importins. Exportins interact with their substrates in the presence of RanGTP in the nucleus and release the cargo in the cytoplasm after GTP hydrolysis [33]. We found exportin 1 (XPO1) which interacts with EIF5A [34], NUP214 [35], NXF3 [36], ORC1 [37], Ran binding protein 2 [38], DDX3 [39], Survivin [40], and telomere reverse transcriptase [41]. XPO1 shuttles between the nucleus and cytoplasm. It is overexpressed in cancer which results in alternate localization of multiple tumor suppressor proteins in the cytoplasm [42].

### Comparison of the ciliary body proteome with aqueous humor and plasma proteomes

**Figure 4:**
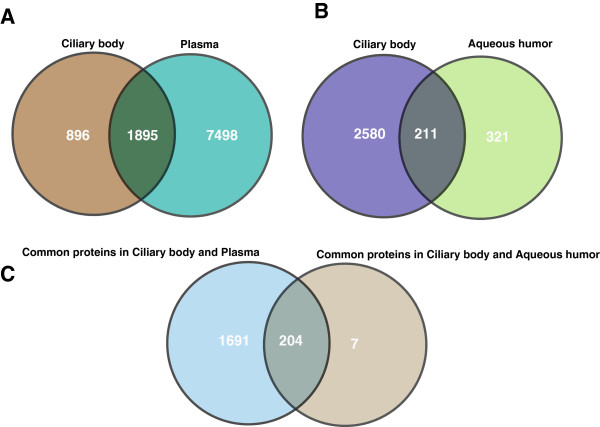
**Comparison of the ciliary body proteome with the aqueous humor and plasma proteome. Panel A** shows comparison of the ciliary body proteins with plasma proteins annotated in the Plasma Proteome Database. **Panel B** depicts comparison of the ciliary body proteome with aqueous humor proteome annotated from the published literature. **Panel C** shows a comparison of proteins that are common to the ciliary body and plasma with those that are common to the ciliary body and the aqueous humor.

The fenestrated ciliary body capillary endothelia allow the flow of blood plasma across the ciliary stroma which helps in the secretion of aqueous humor by the ciliary epithelium. We were interested in proteins derived from ciliary body, which are directly relevant to its physiology, and not those derived from the blood diffusing into the ciliary body. There is a blood aqueous barrier, which permits solutes from the blood vessels of the ciliary stroma into the aqueous humor [11]. We compared the ciliary body proteome with human plasma proteome from Plasma Proteome Database [43] and aqueous humor proteome in order to get the ciliary body specific proteins. A total of 9,393 plasma proteins were compared with the ciliary body proteome and we observed that 896 proteins were unique to the ciliary body proteome as seen in Figure 4A. Proteins detected in the ciliary body were also compared to proteins previously reported in the aqueous humor [7,44-47]. We found 211 proteins that were also reported in the aqueous humor proteome Figure 4B. Only seven of these 211 proteins were described in the plasma (Figure 4C). These unique proteins are *CRYGD* crystallin, gamma D (*CRYGD*), crystallin, gamma S (*CRYGS*) and crystallin, gamma C (*CRYGC*), which maintain the transparency and refractive index of the lens [48,49]. Gamma crystallins have been involved in cataract formation due to aging or mutations. The source of these proteins is likely to be the aqueous humor and not the plasma, as the lens, where these are abundant is an avascular structure receiving all its nutrient supply from the aqueous humor. In addition the aqueous humor removes metabolic waste from the lens. Another molecule is pyruvate kinase muscle (*PKM*), which is involved in glycolysis and serves as a key regulator of energy metabolism in proliferating cells. Frizzled-related protein (*FRZB*) is secreted protein and plays a significant role in the loss of the Wnt signaling pathway in different type of cancers by down regulation of this gene [50]. Ubiquitin fusion degradation 1 (*UFD1L*) forms complex with nuclear protein localization 4 (NPLOC4) and valosin containing protein (VCP). NPLOC4 and VCP are also identified in this study. This complex is required for the degradation of ubiquitinated proteins [51]. Retinoschisin 1 (RS1) plays a significant role in the cellular organization of the retina [44,45].

### Data availability

The raw data derived from the ciliary body proteome is available from several public data repositories. The peptide identifications and MS/MS spectra are available on through Human Proteinpedia (http://www.humanproteinpedia.org) as accession number HuPA_ 00708. The raw data described in this study is freely available from ProteomeCommons.org. Online versions of the data may be found at https://proteomecommons.org/dataset.jsp?i=78277. The data from this study may also be downloaded from Tranche (https://www.proteomecommons.org/tranche/) using the following hash UqPG6uWQU4qG5oAJ9fPxBHNjbvNoBPhyXvoj6T2p4p8VY4S8cNnpeKbpaeROT5diReS2/Wzvbf0e8rGQxWj/yv6jSYUAAAAAAAAClQ== and https://proteomecommons.org/dataset.jsp?i=UqPG6uWQU4qG5oAJ9fPxBHNjbvNoBPhyXvoj6T2p4p8VY4S8cNnpeKbpaeROT5diReS2%2FWzvbf0e8rGQxWj%2Fyv6jSYUAAAAAAAAClQ%3D%3D.

### Gene ontology analysis

**Figure 5:**
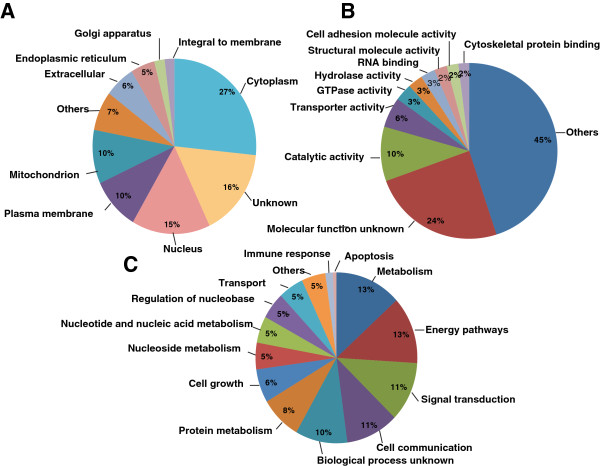
**Subcellular localization and functional annotation of proteins identified from the ciliary body. A**. Gene Ontology analysis for subcellular localization of identified proteins **B**. Molecular function of identified proteins **C**. Biological processes of the identified proteins. The data regarding proteins was obtained from Human Protein Reference Database (http://www.hprd.org).

The identified ciliary body proteins were functionally categorized based on subcellular localization, molecular function and biological processes by searching against the manually-curated Human Protein Reference Database (HPRD; http://www.hprd.org) [52]. The analysis returned only those classifications with at least 2% difference between the annotation terms to limit the number of classifications types. As illustrated in Figure 5A, the majority of the proteins reported in our study were localized to the cytoplasm (27%), nucleus (15%), plasma membrane (10%) or the mitochondria (10%) while 16% of these were unclassified. In the molecular function category, GO terms related to transporter activity are overrepresented. This was expected as the ciliary body secretes aqueous humor by a process of active transport. The majority of the proteins are involved in catalytic activity, GTPase activity, hydrolase activity and structural molecule activity as seen in Figure 5B. A large group of proteins are still unclassified in terms of their molecular function. Moreover, in terms of biological processes, the ciliary body enriched proteins were comprised of a substantially higher percentage of metabolism (22%) and energy pathway (13%) related proteins owing to presence of numerous mitochondria in the inner non-pigmented epithelial layer.

### Biological network analysis

Ingenuity Pathway Analysis was used to facilitate the identification of biological canonical signaling and metabolic pathways. Table 3 depicts the ten most significant pathways enriched by IPA in the ciliary body proteome. In the ubiquitin pathway, one such significant pathway in our results, proteins are tagged for degradation through ubiquitin. The resulting polyubiquitin chain is bound by the proteasome leading to degradation of the tagged protein. The 26S proteasomes are protein complexes of 2 complexes, a 20S core and a 19S regulator that degrade unneeded or damaged proteins by proteolysis. The 20S core is composed of 28 non-identical subunits, 7 alpha subunits, 7 beta subunits and the 19S regulator is composed of 6 ATPase subunits and 12 non-ATPase subunits. This proteasome recognizes polyubiquitin tags attached to protein substrates and initiates the degradation process. In the ubiquitination cascade, E1 can bind with E2s which further bind E3s in a hierarchical way [53] as shown in Additional file 3: Figure S1. In our study, we identified many E1 ubiquitin-like modifier-activating enzymes including UBA1 to UBA7 and MOCS3. We also found E2 ubiquitin-conjugating enzymes and ubiquitin-protein ligase E3A (UBE3A), which helps ubiquitin to attach to a target protein. We also identified deubiquitinating enzymes such as ubiquitin carboxyl-terminal hydrolase 5 (USP5), USP7, USP11, USP14, USP15 which are key regulators of ubiquitin mediated pathways [54]. In the proteosomal family, we reported many proteasomal proteins as listed in Additional file 1: Table S1.

**Table 3:** **Details of Ingenuity Pathways Analysis** (**IPA**) **of top ten canonical pathways**

**Enriched pathways**	**P-value**	**Ratio**
Ubiquitination pathway	2.01E-14	94/263
tRNA charging	2.63E-14	28/38
EIF2 signaling	3.36E-12	68/182
Glycolysis I	1.2E-11	19/23
Gluconeogenesis	4.84E-11	19/24
Valine degradation	5.96E-11	16/18
Ethanol degradation	2.32 E-10	21/30
Caveolar mediated endocytosis signaling	7.56 E-10	35/81
Integrin signaling	1.23 E-09	68/205
TCA cycle	3.32 E-09	17/23

## Conclusions

The ciliary body is a specialized tissue, which has a major role in the formation of the blood-aqueous barrier. It performs many functions such as maintaining a transparent medium inside the eye, nourishing the avascular ocular tissues and maintaining the size and shape of the eye by regulating the intra ocular pressure. By virtue of the unique functions performed by the ciliary body, it is of interest to understand the proteomics profile of this tissue. Our study provides a high resolution mass spectrometric proteome analysis of the ciliary body perhaps identifying the largest set of proteins that appear to be specific to the ciliary body. The information from our study is likely to serve as a baseline for future studies aimed at studying ophthalmic disorders such as glaucoma, uveitis and presbyopia.

## Methods

### Sample collection

The ciliary body samples for the proteomic analysis were obtained at autopsy after obtaining approval from the institutional ethics committee. Clinical details of the donors are documented in Table 4. There was no medical history of glaucoma, other eye diseases or malignancy. The three eye globes were enucleated 3–4 hours post mortem and kept frozen. No eye abnormality was observed by light microscopy. After removal of the cornea, ciliary bodies were excised and stored at −80°C. The ciliary body samples were lysed in 0.5% sodium dodecyl sulfate (SDS), sonicated, homogenized and centrifuged at 13,000 rpm for 15 minutes at 4°C. The supernatant was collected and protein quantitation was carried out by Lowry’s assay (Bio-Rad Hercules, CA; USA). We recovered 2.1, 1.8 and 1.5 mg of proteins from three donor samples.

**Table 4:** Clinical details of donors used in this study

**Sample ID**	**Age (years)**	**Sex**	**Ethnicity**
CB01	80	Female	Indian
CB02	94	Female	Indian
CB03	89	Male	Indian

### *In*-*gel* digestion

The pooled ciliary body samples (~300 μg of protein) were resolved by SDS-PAGE and stained using colloidal Coommassie blue stain. The lane was excised into pieces and destained with 50% acetonitrile in 40 mM ammonium bicarbonate followed by dehydration of the gel pieces with 100% acetonitrile. In-gel reduction was carried out using 5 mM dithiothreitol (60°C for 45 minutes) followed by alkylation using 20 mM iodoacetamide (room temperature for 10 min). These steps reduce the disulfide bonds in proteins and alkylates the free SH groups of Cys residues to yield carbamidomethyl Cys respectively. Removed iodoacetamide and dehydrated the gel pieces by acetonitrile. In-gel digestion was carried out by sequencing grade modified porcine trypsin at a concentration of 10 ng/μl (Promega, Madison, WI, US) in chilled 50 mM ammonium bicarbonate at 4°C to minimize autocatalysis by trypsin and incubated for 45 minutes on ice [55]. Excess trypsin was removed and the gel pieces were immersed in ammonium bicarbonate and incubated overnight at 37°C. The peptides were extracted from the gel bands using 0.4% formic acid in 3% acetonitrile twice, once using 0.4% formic acid in 50% acetonitrile and once using 100% acetonitrile. The extracted peptides were dried using speedvac and stored at −80°C until LC-MS/MS analysis.

### LC-MS/MS analysis

LC-MS/MS analyses of the samples was carried out on a high resolution Fourier transform mass spectrometer, LTQ-Orbitrap Velos (Thermo, Bremen, Germany), as previously described [56,57]. The mass spectrometer was interfaced with Agilent’s 1200 nano-LC system to a trap column (2 cm × 75 μm, C_18_ material 5 μm, 120 Å) and an analytical column (10 cm × 75 μm, C18 material 5 μm, 120 Å). Electrospray source was fitted with an 8 μm emitter tip (New Objective, Woburn, MA) and was applied a voltage of 2000 V. Peptide samples were loaded onto trap column in 3% solvent B (90% acetonitrile in 0.1% formic acid) and washed for 5 minutes. Peptides were eluted using a gradient of 3-35% solvent B for 60 minutes at a constant flow rate of 0.4 μl/min. Xcalibur 2.1 (Thermo Electron, Bremen, Germany) was used for data acquisition. MS spectra were acquired in a data-dependent manner targeting the twenty most abundant ions in each survey scan in the range of m/z 350 to 1,800. The selected ions were excluded for 30s after two MS/MS scans. Target ion quantity for FT full MS and MS2 were 5 × 10^5^ and 2 × 10^5^, respectively. The precursor ion fragmentation was carried out using higher-energy collision dissociation (HCD) using 40% normalized collision energy. The mass spectrometry analysis was carried out with survey scans (MS) acquired at a resolution of 60,000 at 400 m/z and fragment ion scan (MS/MS) acquired at a resolution of 15,000 at 400 m/z.

### Data analysis

The mass spectrometry data analysis was processed using the Proteome Discoverer software (Version 1.3, Thermo Scientific, Bremen, Germany). Mascot and SEQUEST search engines were employed for database searching. The mass spectrometry data was searched against NCBI RefSeq 50 human protein database containing 34,346 sequences with known contaminants. Scans were filtered for - signal to noise ratio of 1.5 and precursor mass range of 300–5000 Da for generation of peak lists. Carbamidomethylation of cysteine was used as the fixed modification and oxidation of methionine as variable modifications. Peptide mass tolerance and fragment mass tolerance were set as 20 ppm and 0.1 Da. We used 1% FDR score cut-off to export the peptide data used for the analysis. GO analysis was carried out using Human Protein Reference Database (HPRD: http://www.hprd.org) [52] and Human Proteinpedia [58] which are GO compliant databases. Pathway analyses were carried out using Ingenuity Pathways Analysis (IPA) software version 7.1 available at http://www.ingenuity.com (Ingenuity Systems, Mountain View, CA, USA). Pathway networks were enriched by IPA with corresponding scores.

## Abbreviations

LC-MS/MS: Liquid chromatography-mass spectrometry; IOP: Intraocular pressure; HPRD: Human Protein Reference Database; OAG: Open angle glaucoma; CAG: Closed angle glaucoma; PSMs: Peptide spectral match; NPCE: Non-pigmented ciliary epithelial layer; PCE: Pigmented ciliary epithelial layer; PSMs: Peptide spectral match; MYH11: Myosin-11; TPM1: Tropomyosin alpha-1 chain; SLC15A2: Solute carrier family 15 member 2; IPA: Ingenuity Pathway Analysis.

## Competing interests

The authors declare that they have no competing interests.

## Authors’ contributions

RG and AP conceptualized the study, designed the experiments and wrote the manuscript. RG carried out the experiments and data analysis. HCH supervised the experiments. KRM provided the ciliary body samples and wrote the manuscript. SMS and AKM were involved in data analysis. SMP and MB was involved in the initial sample processing. DSK and GD edited the manuscript. SSM, KV, TSKP, SC and AP helped to draft the manuscript. All authors read and approved the final manuscript.

## Supplementary Material

Additional file 1: Table S1 A complete list of proteins identified in the ciliary body. This table illustrates reported proteins gene accession, gene symbol, RefSeq accession, protein name, intensity based absolute quantification (iBAQ) score, unique no of peptides, sequence coverage, subcellular localization, molecular function, biological process and domains/motifs. Click here for file

Additional file 2: Table S2 A list of all peptides identified in the ciliary body. This table list the identified peptides in the ciliary body along with protein accession, gene symbol, protein name, Xcorr, Ion score, SEQUEST modifications, Mascot identifications, m/z and delta mass. Click here for file

Additional file 3: Figure S1 Ingenuity Pathway Analysis (IPA) based enrichment of molecular pathway networks. The most significant pathway enriched by IPA in the ciliary body proteome is the ubiquitin pathway. In this cascade, E1 binds with E2s which further bind E3s in a hierarchical way. It results in polyubiquitin chain which leads to degradation of the tagged protein. Proteosomal family also degrade damaged proteins by proteolysis. We reported proteasome subunit alpha type 1 and beta type 1. Ubiquitination and proteasomal degradation is essential for cell cycle, transcription and responses to immune and inflammation. Click here for file
