# Supplementary figures and images for: Characterizing the normal proteome of human ciliary body
Source: Clin Proteomics. 2013 Aug 1;10(1):9. doi: 10.1186/1559-0275-10-9 (PMC3750387; doi:10.1186/1559-0275-10-9)

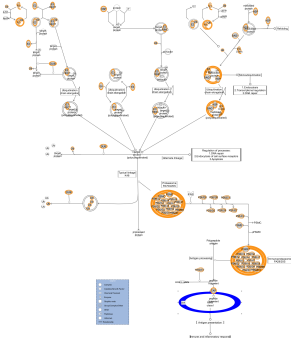

Supplement: Additional file 3: Figure S1 — Ingenuity Pathway Analysis (IPA) based enrichment of molecular pathway networks. The most significant pathway enriched by IPA in the ciliary body proteome is the ubiquitin pathway. In this cascade, E1 binds with E2s which further bind E3s in a hierarchical way. It results in polyubiquitin chain which leads to degradation of the tagged protein. Proteosomal family also degrade damaged proteins by proteolysis. We reported proteasome subunit alpha type 1 and beta type 1. Ubiquitination and proteasomal degradation is essential for cell cycle, transcription and responses to immune and inflammation. [file 1559-0275-10-9-S3.pdf]
